# Diagnostic policies on nephrolithiasis/nephrocalcinosis of possible genetic origin by Italian nephrologists: a survey by the Italian Society of Nephrology with an emphasis on primary hyperoxaluria

**DOI:** 10.1007/s40620-023-01693-x

**Published:** 2023-06-26

**Authors:** Pietro Manuel Ferraro, Chiara Caletti, Giovanna Capolongo, Marco Lombardi, Francesco Scolari, Giuseppe Vezzoli, Corrado Vitale, Giovanni Gambaro

**Affiliations:** 1 UOS Terapia Conservativa Della Malattia Renale Cronica, Fondazione Policlinico Universitario A. Gemelli IRCCS, Università Cattolica del Sacro Cuore, Largo Agostino Gemelli 8, 00168 Rome, Italy; 2 UOC Nefrologia, AOVR, Università degli Studi di Verona, Verona, Italy; 3 Nefrologia e Dialisi, Università della Campania L. Vanvitelli, Naples, Italy; 4 SOC Nefrologia e Dialisi Firenze 2, Ambulatorio Aziendale per la diagnosi terapia e lo Studio della nefrolitiasi, Azienda USL Toscana Ospedale Santa Maria Annunziata, Bagno a Ripoli, Florence, Italy; 5 Spedali di Brescia, UOC Nefrologia, Università di Brescia, Brescia, Italy; 6 UOC Nefrologia, Ospedale San Raffaele, Università Vita e Salute, Milan, Italy; 7 UOC Nefrologia, AO Ordine Mauriziano di Torino, Turin, Italy

**Keywords:** Dialysis, Kidney transplant, Nephrocalcinosis, Primary hyperoxaluria

## Abstract

**Background:**

Primary hyperoxaluria is a genetic disorder of the metabolism of glyoxylate, the precursor of oxalate. It is characterized by high endogenous production and excessive urinary excretion of oxalate, resulting in the development of calcium oxalate nephrolithiasis, nephrocalcinosis, and, in severe cases, end-stage kidney disease and systemic oxalosis. Three different forms of primary hyperoxaluria are currently known, each characterized by a specific enzymatic defect: type 1 (PH1), type 2 (PH2), and type 3 (PH3). According to currently available epidemiological data, PH1 is by far the most common form (about 80% of cases), and is caused by a deficiency of the hepatic enzyme alanine:glyoxylate aminotransferase.

**Methods:**

A survey on rare forms of nephrolithiasis and nephrocalcinosis with a focus on primary hyperoxaluria in the setting of Italian Nephrology and Dialysis Centers, using an online questionnaire, was recently conducted by the Project Group “Rare Forms of Nephrolithiasis and Nephrocalcinosis” of the Italian Society of Nephrology, with the aim of assessing the impact and management of this disorder in clinical practice in Italy.

**Results:**

Forty-five public and private Italian Centers participated in the survey, and responses to the questionnaire were provided by 54 medical professionals. The survey results indicate that 21 out of the 45 participating Centers are managing or have managed primary hyperoxaluria patients, most of whom are on dialysis, or are recipients of kidney transplants.

**Conclusions:**

The data of this survey indicate the need to implement genetic testing in suspected cases of primary hyperoxaluria, not only in the setting of dialysis or transplantation, but also with the aim of encouraging early diagnosis of PH1, which is the only type of primary hyperoxaluria for which specific drug therapy is currently available.

**Graphical abstract:**

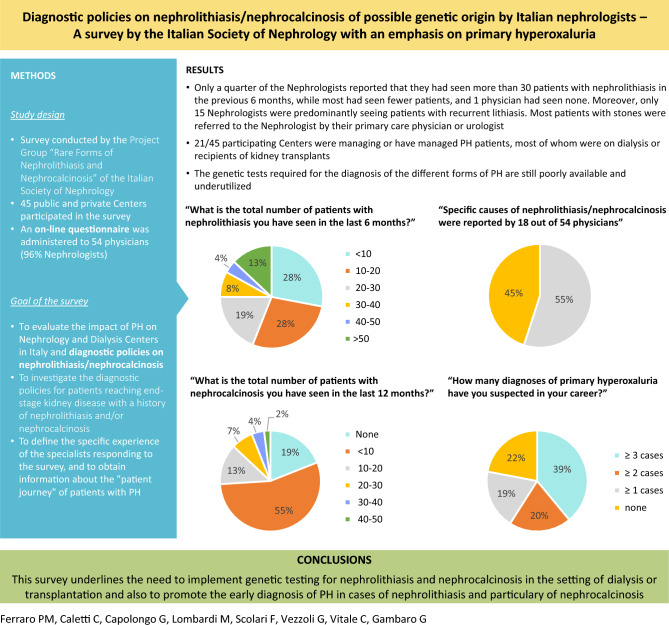

**Supplementary Information:**

The online version contains supplementary material available at 10.1007/s40620-023-01693-x.

## Introduction

Primary hyperoxaluria (PH) is a rare autosomal recessive genetic disorder affecting the metabolism of glyoxylate, the precursor of oxalate [1].

Three different forms of primary hyperoxaluria are currently known, each characterized by a specific enzymatic defect: type 1 (PH1), type 2 (PH2), and type 3 (PH3) [1]. According to currently available epidemiological data, PH1 is by far the most common form (about 80% of cases), and is caused by a deficiency of the enzyme alanine:glyoxylate aminotransferase (AGT), which is localized in hepatic peroxisomes [2]. PH2 is due to a deficiency of glyoxylate reductase/hydroxypyruvate reductase, an enzyme localized in the cytosol of hepatocytes and leukocytes [1]. Finally, PH3 is associated with a mutation in the HOGA1 gene, which encodes 4-hydroxy-2-oxoglutarate aldolase, a mitochondrial enzyme [3].

Primary hyperoxaluria is clinically characterized by high endogenous production and excessive urinary excretion of oxalate, resulting in the development of calcium oxalate nephrolithiasis, nephrocalcinosis, and progression to chronic renal failure and systemic oxalosis (accumulation of calcium oxalate crystals at various sites in the body, including bone tissue, cardiovascular system, and skin). The presence of nephrolithiasis or nephrocalcinosis, usually very severe, predisposes to the development of kidney failure [1].

A survey on rare forms of nephrolithiasis and nephrocalcinosis with a focus on PH in the setting of Italian Nephrology and Dialysis Centers was recently conducted using an online questionnaire, at the initiative of the Project Group “Rare Forms of Nephrolithiases and Nephrocalcinoses” of the Italian Society of Nephrology (SIN), with the aim of assessing the impact of this disorder on clinical practice and management policies in Italy.

## Materials and methods

The SIN questionnaire (see Supplementary Material Online, Table 1) was administered to physicians practicing in public and private Centers all over the country. All participating physicians provided informed consent.

The first section of the questionnaire, named “Nephrology”, was designed to assess the characteristics of referring Centers, with particular emphasis on the clinical burden in the management of conditions such as nephrolithiasis and nephrocalcinosis, where cases of primary hyperoxaluria may “lurk”; its aim was also to investigate the general diagnostic policies applied by the specialists to whom these patients are referred; and, finally, to investigate the specific diagnostic policies for suspected PH.

The second section, named “Dialysis and Transplantation”, aimed to investigate the size of Dialysis Centers and their diagnostic policies for patients reaching end-stage kidney disease (ESKD) with a history of nephrolithiasis and/or nephrocalcinosis.

Finally, the aim of the third section, named “Primary Hyperoxaluria”, was to define the specific experience of the specialists responding to the survey, and to obtain information about the “journey” of patients with PH.

## Results

Four hundred public and private Centers all over the country, were invited to participate in the SIN Survey (see Supplementary Material Online, Table 1); 45 Centers (11.3%) accepted to participate and completed the questionnaire. Responses to the questionnaire were provided by a total of 54 physicians (1 physician in each of 37 Centers, 2 physicians in each of 7 Centers, and 3 physicians in 1 Center). Among the physicians who responded to the questionnaire, 96% were Nephrologists, while 2 physicians (4%) did not report their specialization. The average age among respondents was 51 years (range 29–70).

The responses provided are detailed below.

### First section (nephrology)

**Fig. 1:**
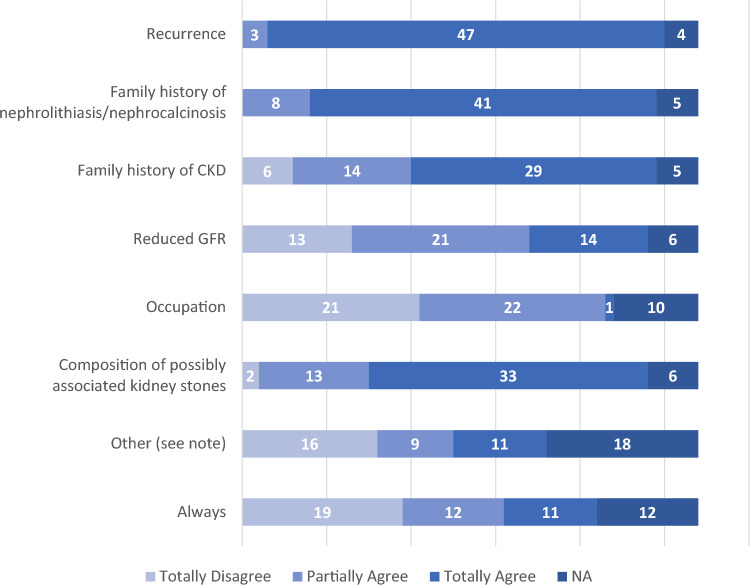
Reasons for the physicians responding to the SIN questionnaire (*n* = 54) to perform a metabolic screening in patients with nephrolithiasis. *The following reasons are specified under 'Other': bilaterality, multi-organ involvement, earliness of onset, stones arising in childhood, staghorn stones and/or stones with potential for infectious complications, genetic factors*

**Fig. 2:**
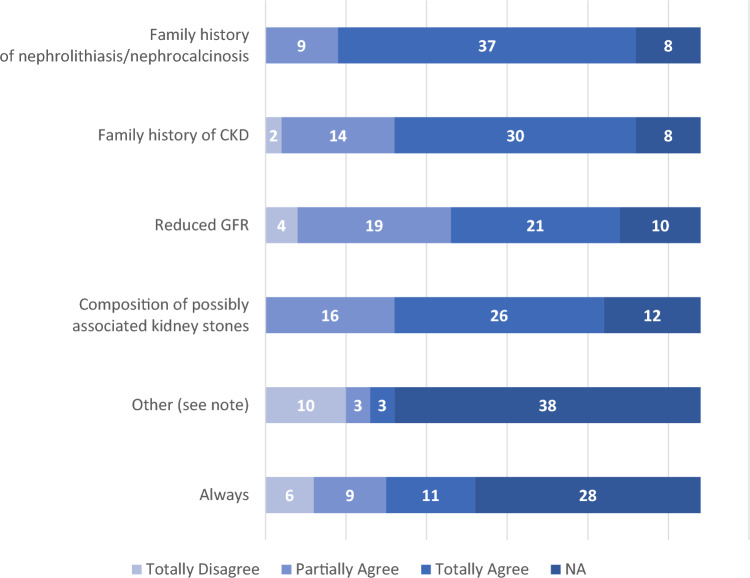
Reasons for the physicians responding to the SIN questionnaire (n = 54) to perform a metabolic screening or second level tests in patients with nephrolithiasis. The following reasons are specified under 'Other': bilaterality, multi-organ involvement, presence of extra-renal signs/symptoms, childhood patients and/or patients with no obvious risk factors for nephrocalcinosis, genetic factors

**Fig. 3:**
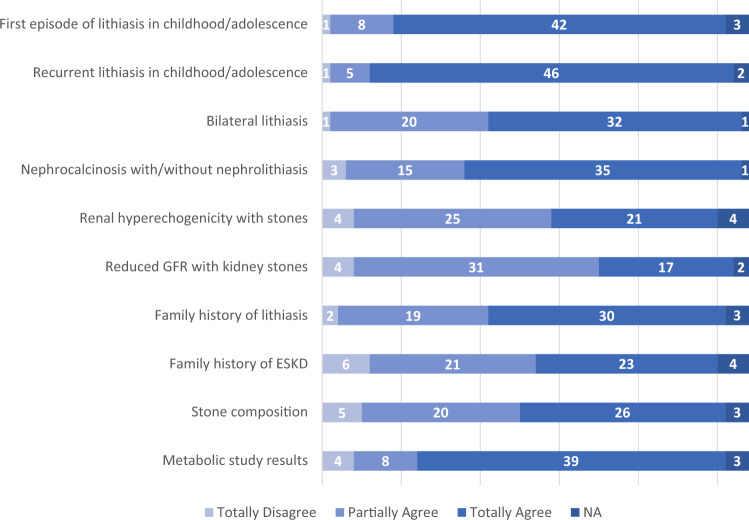
Items of diagnostic suspicion for primary hyperoxaluria in patients with nephrolithiasis/nephrocalcinosis, according to physicians' responses to the SIN questionnaire (*n* = 54)

**Fig. 4:**
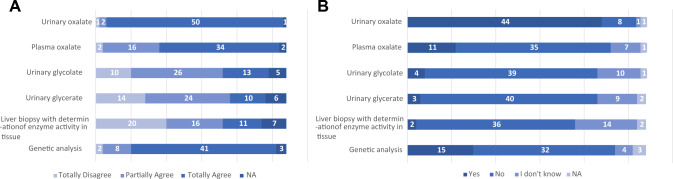
Tests ordered for suspected primary hyperoxaluria in patients with nephrolithiasis/ nephrocalcinosis (**A**) and tests performed in the various Centers (**B**), according to the answers provided by Physicians to the SIN questionnaire (*n* = 54). **A** – What tests would you order for suspected primary hyperoxaluria? **B** – Which of the following tests are performed in your facility?

- **Question 1**: “What is the total number of patients with nephrolithiasis you have seen in the last 6 months?”
- Fifty-four responses were given to this question. The most frequent responses were “Less than 10” (*n* = 15), “Between 10 and 20” (*n* = 15), and “Between 20 and 30” (*n* = 10). Four physicians reported having seen between 30 and 40 patients with nephrolithiasis, 2 physicians between 40 and 50 patients, and 7 physicians more than 50 patients. Only 1 physician reported not having seen any patients with nephrolithiasis in the past 6 months.
- **Question 2**: “How many of these patients had recurrent nephrolithiasis?”
- There were 53 responses to this question (1 missing data). The most frequent responses were “Less than 10%” (*n* = 11) and “Between 10 and 40%” (*n* = 24). Nine physicians responded that between 40 and 80% of the patients seen in the last 6 months had recurrent nephrolithiasis; this percentage was higher than 80% according to 6 physicians. Finally, 3 physicians responded that they had not seen any patients with recurrent nephrolithiasis in the past 6 months.
- **Question 3**: “What is the total number of patients with nephrocalcinosis you have seen in the last 12 months?”
- There were 53 responses to this question (1 missing data). The most frequent responses were “None” (*n* = 10) and “Less than 10” (*n* = 29). Seven physicians reported having seen between 10 and 20 patients with nephrocalcinosis in the past 12 months, 4 physicians between 20 and 30 patients, 2 physicians between 30 and 40 patients, and 1 physician between 40 and 50 patients.
- **Question 4**: “By which department(s) (or specialist) were these patients referred?”
- The majority of patients seen by the surveyed physicians were referred by Urology departments (27%), Primary Care physicians (26%), and Emergency departments (17%). Smaller numbers of patients were referred by other specialty departments including Nephrology (9%), Gastroenterology (7%), Pediatrics (5%), Internal Medicine (5%), Endocrinology (3%), and Medical Genetics (1%).
- **Question 5**: “In your clinical practice, do you perform a metabolic screening in patients with nephrolithiasis?”; and **Question 6**: “For what reasons do you perform a metabolic screening in patients with nephrolithiasis?”.
- Ninety-four percent of physicians routinely carry out metabolic screening for their patients with nephrolithiasis, while only 6% do not consider it necessary. The characteristics of patients with nephrolithiasis undergoing metabolic screening are shown in Fig. 1.
- **Question 7**: “In your clinical practice, do you perform a metabolic screening or second level tests in patients with nephrocalcinosis?”; and **Question 8**: “For what reasons do you perform a metabolic screening or second level tests in patients with nephrocalcinosis?”.
- Eighty-seven percent of physicians regularly carry out metabolic screening or second level tests in patients with nephrocalcinosis, while 13% do not consider it necessary. The characteristics of patients with nephrocalcinosis undergoing metabolic screening or second level tests are shown in Fig. 2.
- **Question 9**: “Have you ever requested genetic testing to clarify the diagnosis in a patient with kidney stones or nephrocalcinosis?”
- Twelve physicians answered “Never”, 15 physicians “Exceptionally”, 13 physicians “Rarely”, and 12 physicians “Often”.
- **Question 10**: “In your experience, what makes (or made) you suspect a primary hyperoxaluria?”
- Based on the answers given, detailed in Fig. 3, the most common items of diagnostic suspicion for primary hyperoxaluria were found to be a first episode of lithiasis in childhood/adolescence, and the presence of recurrent lithiasis in childhood/adolescence.
- **Question 11**: “What tests would you order for suspected primary hyperoxaluria?”; and **Question 12**: “Which of the following tests are performed in your facility?”.
- The physicians’ answers to these questions are detailed in Fig. 4 (Boxes **A** and **B**, respectively).
- **Question 13**: “In case these tests are not available, if you refer to other Centers, please specify for which tests”
- The tests for which the surveyed physicians most often had to refer to other Centers, as part of the diagnostic procedure for PH, were genetic analysis (*n* = 28), plasma oxalate (*n* = 23), and urinary oxalate (*n* = 15). Urinary glycolate (*n* = 11), urinary glycerate (*n* = 10), and liver biopsy with determination of enzyme activity in tissue (*n* = 9) were ordered less frequently.

### Second section (dialysis and transplantation)

**Fig. 5:**
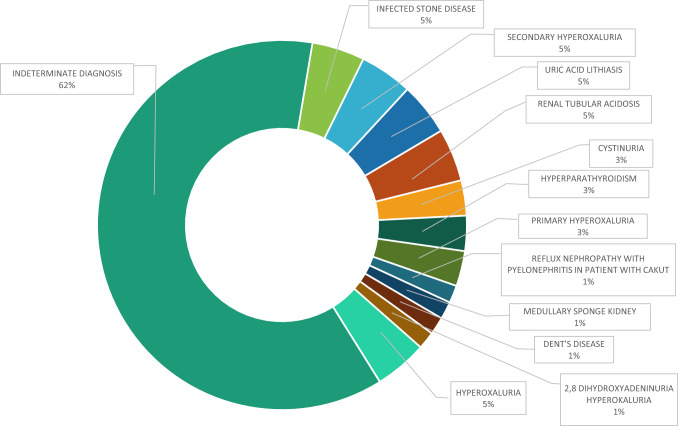
Specified diagnoses in patients on dialysis with nephrolithiasis/nephrocalcinosis, according to the responses provided by the physicians to the SIN questionnaire (*n* = 54). CAKUT = Congenital anomalies of the kidney and urinary tract. ADPKD = autosomal dominant polycystic kidney disease; 2,8-DHA = 2,8-dihydroxyadenine (DHA) nephropathy due to adenine phosphoribosyltransferase deficiency

- **Question 1**: “Does your Center care for patients on chronic dialysis (hemodialysis, peritoneal dialysis)?”; and **Question 2**: “How many dialysis patients does your Center care for?”.
- Ninety-one percent of physicians reported having patients on chronic dialysis in their Center; the average number of dialysis patients per Center was 124 (range 5–400).
- **Question 3**: “How many dialysis patients have a history of recurrent nephrolithiasis or nephrocalcinosis?”; **Question 4**: “How many dialysis patients with a history of nephrolithiasis or nephrocalcinosis have a specific diagnosis of the cause of nephrolithiasis/nephrocalcinosis?”; and **Question 5**: “How many dialysis patients with a history of nephrolithiasis or nephrocalcinosis underwent genetic testing?”.
- Overall, 199 (3.5%) out of 5706 patients on dialysis treatment had a history of recurrent nephrolithiasis/nephrocalcinosis, with an average of 4.5 patients (range 0–22) per Center. Of these, a cause-specific diagnosis of nephrolithiasis/nephrocalcinosis was reported in 30 cases. Genetic testing was performed on a total of 24 dialysis patients with a history of nephrolithiasis/nephrocalcinosis, with an average of 0.6 tests (range 0–5) per Center.
- **Question 5**: “In patients in whom a diagnosis of the cause of nephrolithiasis/ nephrocalcinosis was made, can you specify their diagnoses?”
- Specific causes of nephrolithiasis/nephrocalcinosis were reported by 18 out of 54 physicians (45%); these diagnoses are shown in Fig. 5.

### Third section (primary hyperoxaluria)

**Fig. 6:**
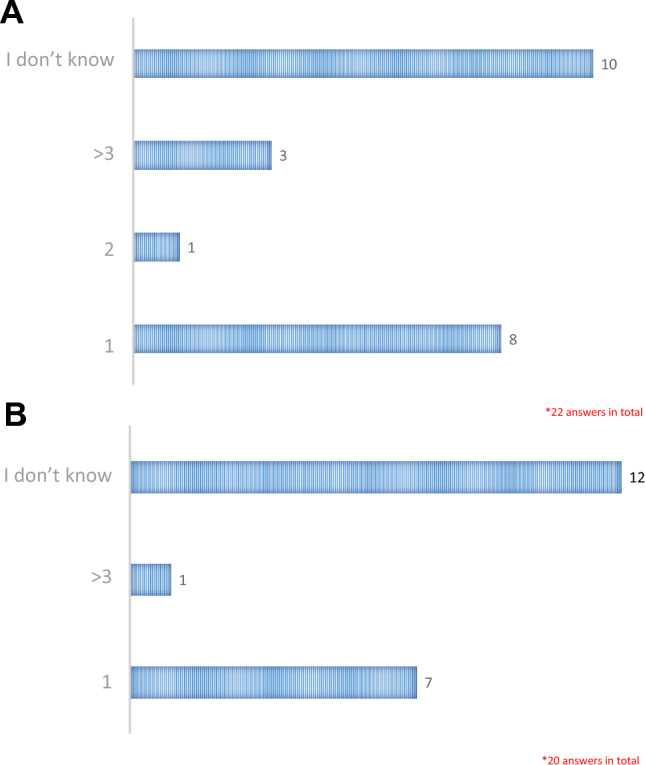
Patients with primary hyperoxaluria who received a double liver-kidney transplant (**A**) or a kidney transplant (**B**), according to the responses provided by the physicians to the SIN questionnaire (n = 22 and n = 20, respectively). **A**. How many patients with primary hyperoxaluria managed at your Center and/or referred to another Center, received a double liver-kidney transplant? **B**. How many patients with primary hyperoxaluria managed at your Center and/or referred to another Center, received a single kidney transplant?

- **Question 1**: “How many diagnoses of primary hyperoxaluria have you suspected in your career?”
- Thirty-nine percent of physicians responded that they had suspected a diagnosis of primary hyperoxaluria in 3 or more cases, 20% in at least 2 cases, and 19% in at least 1 case; finally, 22% of physicians reported that they had never suspected a case of primary hyperoxaluria in their patients.
- **Question 2**: “Does your Center currently manage patients with primary hyperoxaluria?”; and **Question 3**: “Did your Center refer patients with primary hyperoxaluria to another Center in the past?”.
- Out of a total of 45 Centers, 8 answered in the affirmative to Question 2, and 12 to Question 3.
- **Question 4**: “How many patients with primary hyperoxaluria managed at your Center and/or referred to another Center are on dialysis?”
- This question was answered by 19 physicians (35%). The most common answers were “I don't know” (48%) and “1” (37%), while the answers “2”, “3”, and “ > 3” were less common (5% each).
- **Question 5**: “How many patients with primary hyperoxaluria managed at your Center and/or referred to another Center, received a double liver-kidney transplant (7A) or a single kidney transplant (7B)?”.
- Physicians’ responses to these questions are detailed in Fig. 6 (Boxes **A** and **B**, respectively).

## Discussion

The results of this survey in the “Nephrology” section, although based on a non-random sample almost exclusively consisting of Nephrologists (96%), appear to be well representative of the broader European Nephrology community [4]. We therefore assume that the data collected on the clinical management of patients with hyperoxaluria are also representative of the standard practice. Since ninety-six percent of the physicians participating in the survey were Nephrologists, we will refer to them as Nephrologists.

Only a quarter of the Nephrologists reported that they had seen more than 30 patients with nephrolithiasis in the previous 6 months, while most had seen fewer patients, and 1 physician had seen none. Moreover, only 15 Nephrologists were predominantly seeing patients with recurrent lithiasis. Most patients with stones were referred to the Nephrologist by their primary care physician or urologist. These findings confirm what was reported in a survey recently conducted among European Nephrologists and Urologists [4]. Similar considerations can be drawn for the cases of nephrocalcinosis observed by the participating Nephrologists. Overall, these data suggest that there are no true ‘stone Centers’ with a defined organization and coordination between Urologists and Nephrologists, but rather specialists in Nephrology with diagnostic-therapeutic expertise in nephrolithiasis.

The majority of these Nephrologists refer their stone patients for metabolic screening, thus complying with the recommendations of the most important guidelines [5, 6]. This finding, which confirms what has already been observed in Europe [4], is opposite to what has been observed in the United States, where only 15% [7] of stone patients undergo 24-h urine collection, a 'proxy' for metabolic studies. The difference probably lies in the fact that the U.S. study likely investigated a population seen by a mix of specialists (e.g., not only Nephrologists), unlike our survey, which was conducted only among Nephrologists, and the European survey, in which 78.5% of respondents were Nephrologists. Therefore, it is clear that Nephrologists are involved in the management of this disease to determine its causes, but also probably to initiate preventive treatments for stone recurrence, since recurrence is the main criterion for performing a metabolic study. Other criteria frequently considered by Nephrologists to perform a metabolic study include a family history of stone disease and CKD, and the composition of urinary calculi which may suggest the diagnosis. Nevertheless, as we will see below, a definitive diagnosis is not often achieved, and the results of our study suggest that there is still much room for improvement.

A history of recurrent nephrolithiasis/nephrocalcinosis is reported by 3.5% of dialysis patients followed in the Centers of the Nephrologists participating in the survey. This prevalence is similar to that previously found in Italy [8]. We cannot state that recurrent nephrolithiasis/nephrocalcinosis was the cause of ESKD; however, an etiologic diagnosis – that is, a diagnosis that could clarify this point—was made only in a modest number of cases (30/199) (Fig. 5).

The diagnostic suspicion of PH may arise in the presence of recurrent or early-age onset (usually in the first 20 years of life) calcium oxalate nephrolithiasis. Indeed, our survey confirms that the items of diagnostic suspicion for primary hyperoxaluria that most frequently prompt patients with nephropathy/nephrocalcinosis to undergo metabolic screening or second level tests are the onset of a first stone episode in childhood/adolescence and the presence of recurrent lithiasis during childhood/adolescence. However, other conditions (reduced GFR, nephrocalcinosis, metabolic study findings) also receive sufficient attention as possible indicators of hyperoxaluria. This suggests that the suspicion of primary hyperoxaluria is not ignored in adult patients by the Nephrologists participating in the survey. This is particularly important given the fact that, in a significant proportion (about 20 percent) of patients, the condition can remain asymptomatic or paucisymptomatic until adulthood, occurring even relatively later in life [9]. In the large OxalEurope case series, out of 653 patients with PH1 in whom the date of diagnosis was known, it occurred in adulthood in 197 cases (30.2%) [Metry E, *personal communication*].

The first step in the diagnosis of PH is the finding of elevated oxalate levels in the 24-h urine collection, e.g., urine oxalate excretion in excess of 0.46 mmol/1.73 m^2^ per day. Markedly higher urine oxalate levels might increase the clinical suspicion [10, 11], while diagnostic confirmation and differentiation between different types of PH is achieved by biochemical and/or genetic testing [2]. In patients with greatly reduced renal function, plasma oxalate levels are more reliable for diagnosis than urinary levels as well as being predictive of ESKD development [12].

This survey shows that the genetic tests required for the diagnosis of the different forms of PH are still poorly available and underutilized. Only urinary oxalate determination is widely available, while urinary assays of metabolic precursors (glycolate and glycerate) and blood assays of oxalate are even less widely available than genetic testing. It should be remembered that, compared with urinary oxalate levels, the diagnostic utility of serum oxalate concentration has been demonstrated for moderate-to-severe renal impairment, with GFR < 30 mL/min/1.73 m^2^ [13]. Furthermore, serum oxalate is elevated in ESKD [14], although its values are significantly higher in ESKD due to PH [12].

In patients with kidney failure, it has been reported that serum oxalate may increase to levels leading to spontaneous precipitation of calcium oxalate. Thus, one wonders whether the use of genetic testing would be more appropriate when suspecting PH as the cause of CKD/ESKD.

The condition of nephrocalcinosis is not always adequately appreciated as a reason for conducting metabolic screening or second level tests in patients with kidney stones. In fact, there are as many as 15 physicians out of 54 who only partially agree or totally disagree to always investigate it.

This finding is surprising as nephrocalcinosis is a renal parenchymal disease that may or may not be associated with stones but carries a non-negligible risk of ESKD. Moreover, it is frequently an expression of genetic disorders. Nevertheless, only 12 physicians routinely carry out genetic tests in patients with nephrocalcinosis. It is possible that this is due to the small number of laboratories that perform these tests, and to the fact that until now the search for mutations in the involved genes was only available in some Italian and European laboratories. Only recently have the methods of Exon Sequencing in Gene Panels and Whole Exome or Genome Sequencing, which allow the multiple genes involved to be analyzed in a single laboratory test, become more widely available for diagnostic use [15, 16]. Taken together, these data highlight the need for a service that provides Nephrology Centers with access to comprehensive analysis of a panel of genes for nephrolithiasis/nephrocalcinosis.

The results of the SIN survey indicate that a significant number of participating Centers (8 out of 45) currently manage patients with primary hyperoxaluria, mostly on dialysis or with previous hepatorenal or renal transplant. The fact that many Centers refer their patients to other reference Centers is indicative of the need to optimize all steps of case diagnosis and management by referring these patients to physicians with sufficient skills and experience.

The limitations of the present study are primarily due to its retrospective nature since the diagnosis of PH is based on the previous experience of the physicians. In addition, the finding that only 1 of the participating physicians reported not having seen any patients with nephrolithiasis in the past 6 months is indicative of a potential *'selection bias*' in the study, as specialists with an above-average interest/experience in this topic may have responded more frequently. The fact that as many as 21 of the Centers participating in this survey have experience in the management of an ultra-rare condition such as PH should also suggest caution in generalizing the survey results, which may not be representative of the practice patterns of average Adult Nephrology Centers.

Finally, it should be noted that, given the relatively high degree of heterogeneity across countries in terms of policies in place to investigate potential monogenic disorders, future multicenter studies from different countries would bring valuable information to this important aspect of Nephrology.

## Conclusions

The data reported by the survey indicate the need to implement early screening and genetic testing for PH and nephrocalcinosis not only in the setting of dialysis or transplantation, but also to promote early diagnosis of PH in cases of nephrolithiasis and particularly of nephrocalcinosis.

## Supplementary Information

Below is the link to the electronic supplementary material.

Supplementary file1 (DOCX 26 kb)

Supplementary file2 (PDF 96 kb)

## Data Availability

The data underlying this article will be shared on reasonable request to the corresponding Author.
