# Supplementary material for: Diagnostic policies on nephrolithiasis/nephrocalcinosis of possible genetic origin by Italian nephrologists: a survey by the Italian Society of Nephrology with an emphasis on primary hyperoxaluria
Source: J Nephrol. 2023 Jun 26;36(6):1605–14. doi: 10.1007/s40620-023-01693-x (PMC10393840; doi:10.1007/s40620-023-01693-x)
Supplement: Supplementary file 1 — Supplementary file1 (DOCX 26 kb) [file 40620_2023_1693_MOESM1_ESM.docx]

**Table 1. Società Italiana di Nefrologia (SIN) questionnaire for a survey on diagnostic policies on nephrolithiasis/nephrocalcinosis with a focus on primary hyperoxaluria**

| First Section (Nephrology)   1. What is the total number of patients with nephrolithiasis you have seen in the last 6 months? 2. How many of these patients had recurrent nephrolithiasis? 3. What is the total number of patients with nephrocalcinosis you have seen in the last 12 months? 4. By which department(s) (or specialist) are these patients referred? 5. In your clinical practice, do you perform a metabolic screening in patients with nephrolithiasis? 6. For what reasons do you perform a metabolic screening in patients with nephrolithiasis? 7. In your clinical practice, do you perform a metabolic screening or second level tests in patients with nephrocalcinosis? 8. For what reasons do you perform a metabolic screening or second level tests in patients with nephrocalcinosis? 9. Have you ever requested genetic testing to clarify the diagnosis in a patient with kidney stones or nephrocalcinosis? 10. In your experience, what makes (or made) you suspect a primary hyperoxaluria? 11. What tests would you order for suspected primary hyperoxaluria?     1. Which of the following tests are performed in your facility? Urinary oxalate     2. Plasma oxalate     3. Urinary glycolate     4. Urinary glycerate     5. Liver biopsy with determination of enzyme activity in tissue     6. Genetic analysis 12. In case these tests are not available, if you refer to other Centers, please specify for which tests:     - - - 1. Urinary oxalate           2. Plasma oxalate           3. Urinary glycolate           4. Urinary glycerate           5. Liver biopsy with determination of enzyme activity in tissue           6. Genetic analysis |
| --- |
| ***Second Section (Dialysis and Transplantation)***   1. Does your Center care for patients on chronic dialysis (hemodialysis, peritoneal dialysis)? 2. How many dialysis patients does your Center care for? 3. How many dialysis patients have a history of recurrent nephrolithiasis or nephrocalcinosis? 4. How many dialysis patients with a history of nephrolithiasis or nephrocalcinosis have a specific diagnosis of the cause of nephrolithiasis/ nephrocalcinosis? 5. How many dialysis patients with a history of nephrolithiasis or nephrocalcinosis underwent genetic testing? 6. In patients in whom a diagnosis of the cause of nephrolithiasis/ nephrocalcinosis was made, can you specify their diagnoses? |
| ***Third Section (Primary Hyperox***aluria)   1. How many diagnoses of primary hyperoxaluria have you suspected in your career? 2. Does your Center currently manage patients with primary hyperoxaluria? 3. Did your Center refer patients with primary hyperoxaluria to another Center in the past?” 4. How many patients with primary hyperoxaluria managed at your Center and/or referred to another Center are on dialysis? 5. How many patients with primary hyperoxaluria managed at your Center and/or referred to another Center, received a double liver-kidney transplant or a single kidney transplant? |
