# Supplementary figures and images for: Diagnostic policies on nephrolithiasis/nephrocalcinosis of possible genetic origin by Italian nephrologists: a survey by the Italian Society of Nephrology with an emphasis on primary hyperoxaluria
Source: J Nephrol. 2023 Jun 26;36(6):1605–14. doi: 10.1007/s40620-023-01693-x (PMC10393840; doi:10.1007/s40620-023-01693-x)

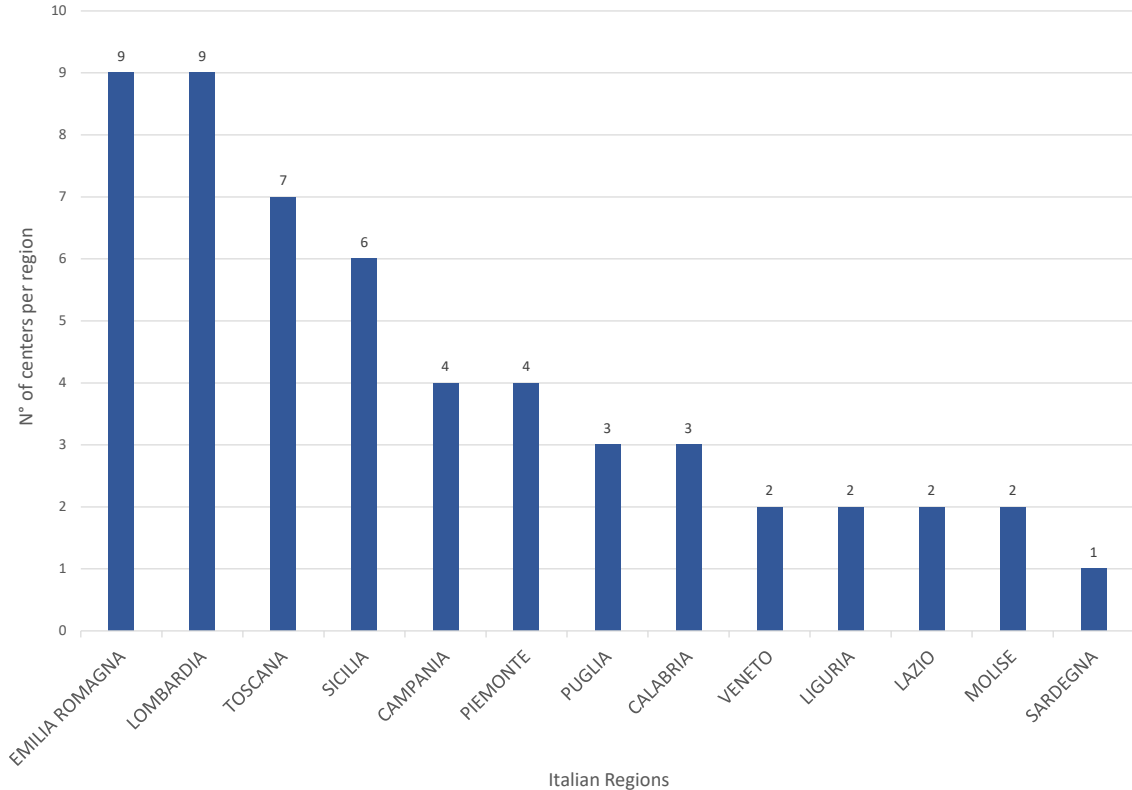

Supplement: Supplementary file 2 — Supplementary file2 (PDF 96 kb) [file 40620_2023_1693_MOESM2_ESM.pdf]
